# Verona Integron-Encoded Metallo-β-Lactamase (VIM)-Producing Pseudomonas aeruginosa Pyelonephritis in a Young Adult: A Case Report

**DOI:** 10.7759/cureus.78932

**Published:** 2025-02-13

**Authors:** Gerson De Freitas

**Affiliations:** 1 Internal Medicine, St. Luke's Hospital, Bethlehem, USA

**Keywords:** carbapenem resistance, pseudomonas aeruginosa, pyelonephritis, vim, vim-pseudomonas

## Abstract

We report a case of pyelonephritis caused by Verona integron-encoded metallo-β-lactamase (VIM)-producing *Pseudomonas aeruginosa* in a 23-year-old female who presented from the community with flank pain and fever, and whose urine culture confirmed the presence of VIM-positive *P. aeruginosa*. Treatment with ceftazidime-avibactam resulted in a favorable outcome. This case highlights the emerging threat of carbapenem-resistant *P. aeruginosa* (CRPA) infections and the effectiveness of ceftazidime-avibactam.

## Introduction

The emergence of carbapenem-resistant *Pseudomonas aeruginosa* (CRPA) is a significant global health concern, particularly due to the occasional production of metallo-β-lactamases (MBLs), such as Verona integron-encoded metallo-β-lactamase (VIM). VIM enzymes hydrolyze nearly all β-lactam antibiotics, including carbapenems; therefore, strains containing the enzyme exhibit resistance to carbapenems and most other β-lactams, severely limiting treatment options [1]. Infections caused by VIM-producing *P. aeruginosa* are associated with increased morbidity, mortality, and healthcare costs, especially in vulnerable populations, such as immunocompromised patients or those with prolonged hospital stays [2,3].

The global prevalence of VIM-producing *P. aeruginosa* varies substantially depending on the region, with regional studies reporting rates between 5% and 30% in hospital settings. In some Mediterranean and Asian countries, prevalence can exceed 40% in certain clinical environments, underscoring the growing concern of antimicrobial resistance [4]. Despite this, VIM-*Pseudomonas* pyelonephritis is not commonly described in the literature, with only rare cases reported [5,6].

The prevalence of VIM-producing *P. aeruginosa* varies geographically, with higher rates reported in Europe, Asia, and parts of Africa. Outbreaks are often linked to hospital environments, particularly intensive care units (ICUs) [7,8].

We describe a case report of a young female with recurrent urinary tract infections (UTIs), who presented with sepsis and pyelonephritis and was found to have VIM-*P. aeruginosa* (VIM-PA) isolated in her urine culture.

## Case presentation

A 23-year-old female presented to the Emergency Department with complaints of right flank pain, fever (102.4°F), and dysuria. She had a history of recurrent UTIs, but no recent hospitalizations or travel, and was most recently treated for a UTI a week ago with ciprofloxacin without improvement.

Physical examination revealed right costovertebral angle tenderness. A complete blood count did reveal a neutrophilic leukocytosis, with a WBC count of 26,000 per microliter (Table 1). Urinalysis showed pyuria with a large presence of leukocytes and innumerable bacteria (Table 2). The patient was empirically started on piperacillin-tazobactam.

**Table 1:** Complete blood count (CBC) with differential WBC - white blood cell; RBC - red blood cell; MCV - mean corpuscular volume; MCH - mean corpuscular hemoglobin; MCHC - mean corpuscular hemoglobin concentration; RDW - red cell distribution width; MPV - mean platelet volume; nRBC - nucleated red blood cell; g - gram; pg - picogram; dL - deciliter; fL - femtoliter; µL - microliter

Parameter	Results	Reference Range
WBC	26	4.31-10.16 Thousand/µL
RBC	3.97	3.81-5.12 Million/µL
Hemoglobin	12.5	11.5-15.4 g/dL
Hematocrit	37.5	34.8 - 46.1%
MCV	94	82 - 98 fL
MCH	33.2	26.8 - 34.3 pg
MCHC	35.3	31.4 - 37.4 g/dL
RDW	14.6	11.6 - 15.1%
Platelet Count	252	149 - 390 Thousand/µL
MPV	9.8	8.9 - 12.7 fL
nRBC	0	/100 WBCs
Differential		
Segmented %	79	43 - 75%
Lymphocytes %	16	14 - 44%
Monocytes %	4	4 - 12%
Eosinophils %	1	0 - 6%
Basophils %	0	0 - 1%
Immature Grans %	0	0 - 2%
Absolute Immature Grans	0	0.00 - 0.20 Thousand/µL
Absolute Neutrophils	1.85	1.85 - 7.62 Thousand/µL
Absolute Lymphocytes	0.89	0.60 - 4.47 Thousand/µL
Absolute Monocytes	0.64	0.17 - 1.22 Thousand/µL
Absolute Eosinophils	0.05	0.00 - 0.61 Thousand/µL
Absolute Basophils	0	0.00 - 0.10 Thousand/µL

**Table 2:** Urinalysis N/A - not applicable; RBC - red blood cell; WBC - white blood cell; mg - milligram; dL - deciliter

Parameter	Result	Reference Range
Color	Yellow	N/A
Clarity	Turbid	N/A
Specific Gravity	1.025	1.003 - 1.030
Glucose	Negative	Negative, mg/dL
Ketone	Trace	Negative, mg/dL
Blood	Trace	Negative
Nitrite	Negative	Negative
Leukocytes	Large	Negative
pH	5.5	4.5 - 8.0
Protein	Negative	Negative
Bilirubin	Negative	Negative
Urobilinogen	<2.0	<2.0 mg/dL
RBC	Negative	Negative
WBC	Innumerable	Negative
Epithelial Cells	Negative	Negative
Bacteria	Innumerable	Negative
Mucus Threads	Negative	Negative

Despite initial treatment, the patient's fever persisted. Urine culture yielded >100,000 colony-forming units per milliliter of multidrug-resistant *P. aeruginosa*. Antimicrobial susceptibility testing revealed resistance to carbapenems, fluoroquinolones, and aminoglycosides, but susceptibility to ceftazidime-avibactam.

Treatment was switched to ceftazidime-avibactam (2.5 g IV every eight hours). The patient showed clinical improvement within 48 hours of starting ceftazidime-avibactam, with improvement in pain, fever, and WBC count. She completed a 14-day course of treatment and was discharged with a resolution of symptoms. Follow-up urine culture, at two weeks post-treatment, was negative.

## Discussion

The VIM was initially reported from Italy in 1999 [9]. The literature reveals multiple reported variants. VIM enzymes mostly occur in *P. aeruginosa*, also *Pseudomonas putida*, and, less commonly, in *Enterobacteriaceae*. This group of enzymes typically hydrolyzes all beta-lactams except monobactams and evades all beta-lactam inhibitors [10].

VIM-producing *P. aeruginosa* represents an emerging concern in clinical infectious disease management. Epidemiological data suggest increasing global dissemination, with the highest prevalence observed in Southern European and Asian healthcare systems [11].

The prevalence of VIM-producing *P. aeruginosa* varies geographically, with higher rates reported in Europe, Asia, and parts of Africa. Outbreaks are often linked to hospital environments, particularly ICUs, where contaminated water systems and medical devices serve as reservoirs. In Europe, the prevalence ranges from 0.4% in Spain to 12.6% in Italy among carbapenem-resistant isolates [7,8,12]. A systematic review in Iran reported a pooled prevalence of 13% for VIM-1-positive *P. aeruginosa* strains [13].

Pyelonephritis caused by VIM-producing *P. aeruginosa* is rare, with only a few documented cases in the literature. Previously reported cases typically involved nosocomial acquisition, whereas our patient presented with a community-onset infection [5,6].

In regard to the treatment of VIM-*Pseudomonas*, ceftazidime-avibactam has been used both alone and in combination with monobactams such as aztreonam as therapeutic alternatives [14,15]. In our particular case, the patient did well with ceftazidime-avibactam monotherapy.

Our case adds to the limited literature on community-acquired VIM-producing *P. aeruginosa* pyelonephritis. The successful use of ceftazidime-avibactam monotherapy underscores its efficacy against MBL-producing pathogens, particularly when combined with early detection and appropriate antimicrobial stewardship, based on cultures and sensitivities.

## Conclusions

The increasing global prevalence of VIM-producing *P. aeruginosa* demands continued vigilance and innovative therapeutic strategies. This case report illustrates the successful management of a patient with pyelonephritis caused by VIM-PA using ceftazidime-avibactam monotherapy. It emphasizes the importance of considering resistant pathogens, even in community-acquired infections. Continued surveillance and judicious use of antibiotics are crucial to prevent the further spread of these resistant organisms.
